# Rescue for an advanced aging patient with synchronous AOSC and gallstone ileus: a case report and literature review

**DOI:** 10.3389/fsurg.2025.1685238

**Published:** 2025-11-18

**Authors:** Junhui Wu, Jun Lu, Zhong Jia

**Affiliations:** 1 The Fourth School of Clinical Medicine, Zhejiang Chinese Medical University, Hangzhou First People’s Hospital, Hangzhou, China; 2 Department of Hepatobiliary and Pancreatic Surgery, Affiliated Hangzhou First People’s Hospital, School of Medicine, Westlake University, Hangzhou, Zhejiang, China

**Keywords:** acute obstructive suppurative cholangitis, cholecystogastric fistula, cholecystolithiasis, gallstone ileus, percutaneous transhepatic cholangial drainage, surgery

## Abstract

**Background:**

Cholecystolithiasis is the most common disease of the gallbladder. Both acute obstructive suppurative cholangitis (AOSC) and gallstone ileus are critical clinical conditions requiring urgent intervention. However, their synchronous occurrence, particularly in elderly patients, presents a significant therapeutic challenge. In such scenarios, an optimal treatment strategy is essential to ensure patient safety while minimizing procedural risks.

**Case presentation:**

Herein, we described a 91-year-old women with cholecystolithiasis who was admitted for a day of abdominal pain accompanied by jaundice and fever. Upon admission, the patient was hemodynamically instable, and blood tests showed elevated white blood cell count and severe liver dysfunction. Emergency computed tomography (CT) revealed intra- and extra-hepatic bile duct dilation with pneumobilia, sludge-like stone at the distal common bile duct (CBD), a cholecystogastric fistula, and a gallstone within the gastric lumen. Soon after, the patient suffered from periumbilical pain. Re-evaluation CT showed the gastric gallstone had migrated into the intestinal lumen, causing gallstone ileus. We first performed ultrasound-guided percutaneous transhepatic cholangial drainage. Three days later, the symptoms resolved. We subsequently performed a curative surgery, including enterolithotomy, cholecystectomy, CBD exploration, and fistula closure. After surgery, the patient recovered successfully. At 3 months of follow-up, she resumed daily activities, with no adverse events.

**Conclusions:**

Synchronous AOSC and gallstone ileus can be life-threatening; however, AOSC carries a higher mortality risk and should be addressed as the immediate priority. In hemodynamically unstable patients, particularly the elderly, extensive surgery should be avoided in the acute phase to reduce perioperative risk. Once stabilized, enterolithotomy and definitive repair can be performed to achieve a favorable outcome.

## 1 Introduction

Cholecystolithiasis is one of the most common biliary diseases, but its complications sometimes manifest as rare and life-threatening emergencies. Acute obstructive suppurative cholangitis (AOSC) is a severe clinical condition, and if not promptly treated, its mortality rate can reach 80%–90% (1). Gallstone ileus, caused by large gallstones entering the gastrointestinal tract through a biliodigestive fistula, occurs in approximately 0.3%–0.5% of patients with cholelithiasis (2), with a mortality rate of 12%–27% (3). To our knowledge, synchronous AOSC and gallstone ileus have not been previously reported in the literature. This condition is particularly rare and poses significant challenges in elderly frail patients with hemodynamic instability. Herein, we report a case of a 91-year-old female patient who simultaneously suffered from synchronous AOSC and gallstone ileus caused by a fallen gallstone. Through a staged treatment approach, we successfully saved the patient's life. This treatment strategy provides valuable clinical experience and practical guidance for managing similar high-risk patients.

## 2 Case presentation

**Figure 1:**
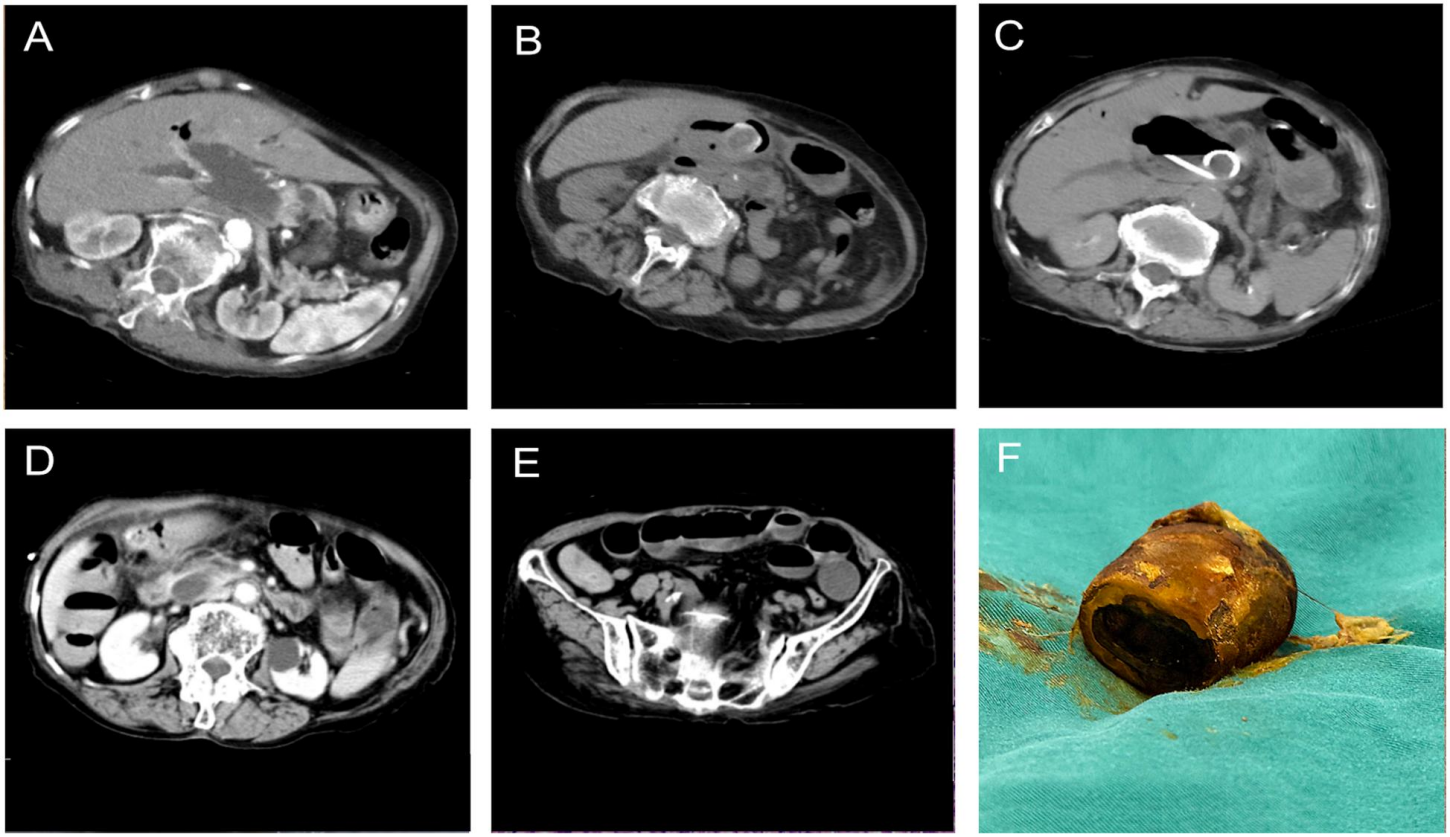
Computed tomography revealed dilation of the common bile duct with pneumobilia **(A)**. Computed tomography revealed a suspected cholecystogastric fistula with an ectopic gallstone located in the gastric antrum **(B)**. Computed tomography revealed the percutaneous transhepatic cholangial drainage was placed in the dilated common bile duct **(C)**. Re-evaluation computed tomography revealed the gallstone in the gastric lumen had disappeared **(D)**. Computed tomography revealed an impacted radiopaque gallstone accompanied by its proximal bowel loop dilation, consistent with gallstone ileus **(E)**. The surgically removed gallstone was hard in texture, oval in shape, and yellowish-brown in color, measuring 3.3 cm in maximum diameter **(F)**.

A 91-year-old woman (BMI 20.5) with a history of chronic cholelithiasis presented with acute abdominal pain, jaundice, and high fever of one day's duration. Over the past three years, she had undergone multiple nonsurgical interventions, including percutaneous transhepatic gallbladder drainage and repeated antibiotic therapy. During the last year, the frequency of acute abdominal pain episodes increased, although she had no other comorbidities or relevant medical history. On examination, the patient was in a passive posture with an apathetic facial expression, hypotensive (80/40 mmHg), febrile (39°C), jaundiced, with reduced bilateral handgrip strength and marked right upper quadrant tenderness. Laboratory tests revealed leukocytosis (20,000/μL), hyperbilirubinemia (total bilirubin: 64 μmol/L; direct bilirubin: 36 μmol/L), and elevated liver enzymes (alanine aminotransferase: 389 U/L; aspartate aminotransferase: 1,175 U/L). Emergency computed tomography (CT) revealed intra- and extrahepatic bile duct dilatation with pneumobilia (Figure 1A), a sludge-like stone in the distal common bile duct (CBD), a cholecystogastric fistula, and a gallstone within the gastric lumen (Figure 1B). After multidisciplinary discussion, the patient underwent emergency percutaneous transhepatic cholangial drainage (PTCD) combined with antibiotic therapy (Figure 1C). Her condition stabilized within three days. Unexpectedly, she subsequently developed periumbilical abdominal pain. Repeat CT revealed disappearance of the gastric gallstone (Figure 1D) and an ectopic gallstone impacted in the ileal lumen with proximal bowel loop dilatation (Figure 1E). Due to the large size of the gallstone, early surgical intervention was undertaken, including enterolithotomy (Figure 1F), cholecystectomy, CBD exploration, and fistula closure. The total operative time was approximately 40 min. The PTCD tube was left *in situ* to function as a T-tube. Pathological analysis confirmed a mixed-composition gallstone measuring 3.3 cm in diameter. The postoperative course was uneventful apart from transient acidosis related to mild abdominal infection. As the patient refused discharge with the drainage tube in place, she remained hospitalized until successful PTCD tube removal thirty days later, and was then discharged in stable condition. At three months of follow-up, the patient had fully recovered without recurrence or adverse events.

## 3 Discussion

Bouveret's syndrome results from large gallstones passing through a biliodigestive fistula and causing gastric outlet obstruction (4). When gallstones pass beyond the gastric outlet into the small intestine, they may lead to gallstone ileus. The radiological diagnosis of gallstone ileus is traditionally based on Rigler's triad, which includes radiopaque gallstones, pneumobilia, and bowel loop dilatation. The presence of two of these three findings is considered diagnostic, while the additional demonstration of a change in gallstone position on follow-up imaging is termed Rigler's tetrad (5). Gallstone ileus is an uncommon cause of mechanical bowel obstruction, accounting for approximately 1%–4% of all cases (6). However, among patients over 65 years of age, it has been reported to account for up to 25%, reflecting both the higher prevalence of cholelithiasis and the frequent delays in diagnosis within this population (7). Without timely and appropriate intervention, gallstone ileus is associated with a mortality rate of up to 30% (8). In this case, the diagnosis was straightforward based on the patient's typical clinical presentation and characteristic imaging findings. However, synchronous AOSC and gallstone ileus are extremely rare. In elderly and frail patients, the treatment process is particularly challenging, requiring dynamic assessment of the clinical condition and careful balance between surgical risks and disease control.

In formulating the treatment plan, we conducted a personalized assessment of the patient, focusing on her overall condition, medical history, and perioperative risks. Frailty, as a key predictor of perioperative outcomes in elderly patients, is typically assessed using various tools. Common frailty assessment tools include the Clinical Frailty Scale, Frailty Index, and Frailty Phenotype (9). These tools evaluate different physiological and functional indicators, such as physical activity, gait speed, muscle strength, cognitive function, and health deficits, helping clinicians gain a comprehensive understanding of the patient's frailty level (9). They effectively identify frail patients and support the development of a personalized treatment plan. However, considering the clinical convenience and timeliness, we chose handgrip strength measurement as the simplest and most efficient method for assessment. Studies have shown that handgrip strength is an effective reflection of frailty and is closely related to postoperative outcomes and hospital stay duration (10). Handgrip strength below 25% of body weight is significantly associated with adverse postoperative outcomes (11). Through this measurement, we were able to quickly assess the patient's frailty and, combined with her advanced age, hemodynamic instability, and acute symptoms, further confirmed her frailty. Therefore, in this case, we prioritized life-saving interventions and adopted a staged treatment strategy to reduce perioperative risks. In the first stage, based on a comprehensive assessment of the patient, we did not choose endoscopic retrograde cholangiopancreatography or emergency surgery, as these treatment options posed higher risks given the patient's frail condition and hemodynamic instability. Simultaneously, considering the severity of the symptoms and high infection risk, we also did not choose conservative management, as it could not effectively and promptly alleviate the biliary obstruction. Consequently, we urgently performed ultrasound-guided PTCD to stabilize hemodynamics and improve impaired liver function.

The decision regarding emergency surgery should take into account three critical factors. (A) Gallstone characteristics: The ileocecal valve represents the key anatomical barrier. Gallstones ≥3 cm in maximum diameter are unlikely to pass spontaneously, whereas stones <2.5 cm may pass through the gastrointestinal tract without obstruction (7, 12). However, discrepancies between the apparent size on imaging and the actual diameter may occur due to cross-sectional angle, leading to underestimation. As in this case, CT imaging showed an impacted enteric gallstone measuring 2.4 cm, whereas postoperative pathology revealed an actual diameter of 3.3 cm. Evidently, the imaging assessment underestimated the true size of the stone. Therefore, in clinical practice, clinicians should remain highly vigilant for potential misjudgment arising from such discrepancies. Therefore, clear communication with patients is essential. In addition, large round gallstones tend to be more difficult to pass compared with irregularly shaped ones. (B) Patient's general condition: In patients who are unfit for surgery, conservative measures such as oral administration of lubricating agents (e.g., cooking oil or liquid paraffin) may be considered. If the ectopic gallstone is located in the stomach or duodenum, mechanical lithotripsy under endoscopic guidance may be attempted, although the success rate is only about 10% and carries a risk of gastrointestinal perforation (13). In this case, the patient was frail and hemodynamically unstable on admission; therefore, we initially focused on managing AOSC. During this process, the gallstone migrated, resulting in a missed optimal window for endoscopic removal. In addition, considering the potential risk of gastrointestinal perforation, the patient initially refused endoscopic extraction. (C) Comorbidities: For example, diabetes may increase the risk of postoperative infection. Although spontaneous passage of gallstones into the feces has been reported, gallstones ≥2 cm frequently result in intestinal obstruction (14). Therefore, surgical management is generally recommended for gallstone ileus (5, 14). We ultimately decided to perform surgical intervention for this patient because CT imaging revealed a gallstone measuring 2.4 cm in diameter that had already caused intestinal obstruction. Early intervention could prevent further aggravation of gallstone ileus and the associated pathophysiological changes, thereby reducing the risk of complications and improving prognosis.

For low-risk patients, a one-stage procedure consisting of enterolithotomy, cholecystectomy, and fistula closure may be performed. In contrast, for high-risk patients (e.g., those with severe comorbidities, hemodynamic instability, or technically challenging surgical dissections), enterolithotomy alone or a two-stage approach—initial enterolithotomy followed by delayed cholecystectomy and fistula closure—may be more appropriate (14). Reported mortality rates are 11.7% for enterolithotomy alone and 16.9% for the one-stage procedure (15). Therefore, enterolithotomy is generally recommended as the preferred treatment. However, Kirchmayr et al. and Alencastro et al. supported one-stage procedures in selected patients with favorable operative conditions, demonstrating acceptable outcomes (16, 17). Chuah et al. further compared one- and two-stage strategies in a case series, showing that individualized decision-making based on perioperative status is crucial (18). We share the same perspective as the above studies, and believe that surgical risks should be dynamically assessed according to each patient's specific condition. Such real-time evaluation is more accurate and appropriate. For some high-risk patients, overall status may improve after a series of effective supportive and interventional treatments, allowing them to transition into a lower-risk group suitable for surgery. In this case, we chose to perform a one-stage procedure. This decision was based on several factors: (A) The patient responded well to PTCD, which successfully relieved the biliary obstruction, significantly improved hemodynamics and liver function, and led to rapid clinical symptom resolution, creating favorable conditions for the subsequent surgery; (B) The effective drainage by PTCD and the management of antibiotics improved the intraoperative visibility and conditions, and there was no significant adhesion between the gallbladder and surrounding tissues, thus reducing the difficulty of the surgery and the risk of complications; (C) Although a two-stage procedure can reduce risks, it would increase the patient's burden of undergoing two surgeries and potentially delay the treatment of gallbladder stones, which could increase the risk of recurrence or malignant transformation.

In recent years, perioperative optimization strategies have become increasingly important in the surgical management of frail elderly patients. Preoperative optimization, through systematic interventions before surgery, can significantly enhance physiological reserve and surgical tolerance. Multiple guidelines and clinical studies have demonstrated that comprehensive prehabilitation should address several domains, including nutritional support, physical training, respiratory exercises, and optimization of medical comorbidities. Nutritional interventions (e.g., protein and caloric supplementation and correction of malnutrition) have been shown to effectively reduce postoperative infections and complications (19). Physical training (e.g., endurance and resistance exercises) and respiratory training (e.g., balloon-blowing, inspiratory resistance devices) improve cardiopulmonary function and muscle strength, thereby enhancing the patient's ability to tolerate surgical stress (20). In addition, active management of cardiovascular, respiratory, and metabolic diseases, correction of anemia and electrolyte imbalances, and appropriate adjustment of medications help stabilize the patient's physiological status during the perioperative period (21). Intraoperatively, minimally invasive techniques, reduced operative time and blood loss, careful fluid management, and tailored anesthesia can minimize surgical trauma and perioperative risk in frail elderly patients (22). Postoperatively, individualized implementation of Enhanced Recovery After Surgery principles is emphasized. Key elements include early oral intake and mobilization, effective pain and delirium management, pulmonary rehabilitation, and continued nutritional and physical support (23–25). These interventions help reduce complications, shorten hospital stay, and improve functional recovery, even in frail or high-risk elderly populations. Therefore, systematically incorporating frailty screening and multimodal perioperative optimization strategies into the management of elderly patients with complex biliary emergencies can enhance surgical safety and improve overall clinical outcomes.

Finally, several practical tips for perioperative management should be highlighted: (A) If the CBD is not markedly dilated, it is preferable to replace the PTCD tube with a T-tube during surgery. Otherwise, the PTCD tube should be preserved for at least one month postoperatively. (B) The optimal timing for endoscopic mechanical lithotripsy is often missed. Therefore, if an impacted gallstone is located in the duodenum, it is preferable to push it into the proximal small bowel to a distance of at least 30 cm beyond the ligament of Treitz, thereby reducing the risk of a high-position fistula (12). This, however, is not an absolute requirement. If the gallstone cannot be advanced, the duodenum may be opened directly, and the stone removed. Closure should be performed with a double-layer technique (mucosa-to-mucosa followed by serosa-to-serosa suturing) or with a “vertical incision and horizontal suture” method. (C) Fistula resection may be considered when the patient's condition allows. If the gastric opening of the fistula is <3 cm, it can usually be closed directly. Larger fistulas should be reinforced with a patch of greater omentum, similar to gastric perforation repair (13). (D) In elderly patients with limited life expectancy, asymptomatic biliodigestive fistulas do not require surgical intervention. Major surgery should be avoided unless a life-threatening risk is imminent or unavoidable. (E) The maximum diameter of the impacted gallstone should not be judged based on a single imaging study. All available imaging data, especially the original gallbladder stone size, should be carefully compared. For example, in our case, the gallstone measured 3.3 cm on pathology, which was larger than the 2.4 cm diameter observed on CT. Fortunately, early surgical intervention was undertaken. In summary, the key to successful management lies in comprehensive evaluation and an individualized therapeutic strategy tailored to the patient's condition.

## 4 Conclusion

Early recognition of synchronous AOSC and gallstone ileus in elderly patients is crucial, as timely intervention can be life-saving. Therapeutic strategies must balance operative risks with the need for definitive disease control. This case demonstrates the critical role of a staged treatment approach, prioritizing life-saving interventions, particularly in frail and hemodynamically unstable patients, which is key to reducing perioperative risks. Subsequently, timely definitive surgery was performed, ensuring the best therapeutic outcomes and prognosis. Therefore, an individualized treatment plan, adapted to the patient's evolving clinical status and continuously assessed in real time, is essential for providing optimal care in complex biliary emergencies.

## Data Availability

The datasets presented in this article are not readily available because of ethical and privacy restrictions. Requests to access the datasets should be directed to the corresponding author.
